# Detection of Quality Deterioration of Packaged Raw Beef Based on Hyperspectral Technology

**DOI:** 10.1002/fsn3.70022

**Published:** 2025-03-19

**Authors:** Cheng Wu, Yingjie Feng, Jiarui Cui, Zhang Yao, Hailong Xu, Songlei Wang

**Affiliations:** ^1^ School of Food Science and Engineering Ningxia University Yinchuan China; ^2^ Yinchuan Hi‐Tech Industrial Development Zone Yinchuan China

**Keywords:** chemometrics, hyperspectral imaging, malondialdehyde, optimization algorithm, packaged beef, visualization

## Abstract

It is an important measure to ensure food quality and safety that real‐time monitoring of the key quality indicators of fresh meat after packaging in the process of storage and transportation. The feasibility of combining hyperspectral imaging (HSI) technology with chemometrics and deep learning to detect the quality deterioration of polyethylene (PE)‐packaged raw beef, especially for a key lipid oxidation indicator of malondialdehyde (MDA) content, was explored in this study. The feasibility of filtering to overcome the interference of packaging film on the spectral data was further investigated. Near‐infrared HSI (400–1000 nm) was used to collect spectral and spatial data of beef samples during short‐term storage. A least squares regression (PLSR) and echo‐neural network optimized by vulture optimization algorithms (BES‐ESN) models were developed by multivariate data processing methods. The results showed that the performance of models established by PE‐packed beef samples was usually inferior to that established by unpacked beef samples. The changes of MDA content in beef were visualized according to the optimal model. In addition, Gaussian filtering was applied to reduce the interference effect caused by the packaging film. The results have demonstrated that HSI combined with Gaussian filter preprocessing and multivariate data processing provided an efficient and reliable tool for detecting the freshness of beef in PE packaging. The best model had a coefficient of determination (*R* ^2^_P_) of 0.8309 and a root mean squared error of prediction (RMSEP) of 0.2180, demonstrating the potential of hyperspectral techniques for real‐time monitoring of packaged raw meat quality. The findings can provide some references for the meat industry to develop advanced non‐invasive quality assurance systems in the meat industry.

## Graphical abstract

A rapid nondestructive detection of fresh meat after packaging based on hyperspectral imaging was proposed. Gaussian filtering was applied to reduce the interference effect caused by the packaging film. Multivariate combinatorial modeling based on echo‐neural networks optimized by vulture optimization algorithms.

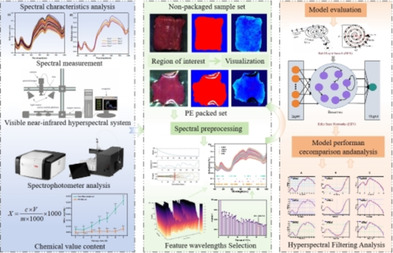

## 1 Introduction

With the improvement of living standards, consumers are paying more attention to diet health and product quality, especially the demand for high‐quality beef, which has risen significantly (Dong et al. 2022). Most beef sold in the market is in the form of cold meat, which is susceptible to environmental, technical, and transportation factors, resulting in contamination and deterioration, so as to affect food safety. As an important food protection technology, vacuum packaging can effectively extend the shelf life of fresh meat by removing air and sealing it to block oxygen and microbial invasion (Zhang, Pan, and Lu 2023). Polyethylene (PE) packaging is a widely used vacuum packaging material known for its durability, transparency, and ability to minimize oxygen exposure, thereby preserving the freshness of perishable foods.

Monitoring key quality indicators of packaged raw meat during storage and transportation is an important part of ensuring food safety (Antequera et al. 2021). Fat oxidation is one of the main factors affecting the decline in meat freshness, second only to microbial changes. It not only leads to deterioration of meat texture, odor, and rancidity, which affects the freshness and nutritional value of meat, but also promotes the oxidation of myoglobin in meat (Cheng et al. 2023). The malondialdehyde (MDA) is a common lipid oxidation product in meat and other foods, and it is often used as an indicator of the degree of lipid oxidation in foods (Wang et al. 2019). Therefore, a comprehensive analysis of beef quality is of great value and significance in maintaining high standards and sustainable development of the meat industry.

Traditional beef quality detecting methods mainly include high‐performance liquid chromatography (HPLC), gas chromatography (GC), and mass spectrometry (MS) techniques. Despite their high resolution, these methods are complex and time‐consuming to operate, making it difficult to meet the urgent needs of meat suppliers for efficient and non‐invasive detecting techniques (Feng et al. 2024). With the increasing demand for ease of operation and sample non‐destructiveness, non‐destructive detecting techniques have received increasing attention in the field of meat quality assessment.

Hyperspectral imaging (HSI) technology can simultaneously acquire the spectral and spatial information of the samples, which combines the advantages of imaging and spectral analysis and has the characteristics of being fast, non‐destructive, and noncontact (Cui, Li, et al. 2024). It has been widely used in the detection of meat quality, such as beef, pork, and chicken, as well as agricultural products like fruits, vegetables, and grains. However, the current research on beef quality detection based on HSI technology mostly focuses on the inspection of non‐packaged beef products, which increases the workload of sample processing before detection (Jo et al. 2024). In order to ensure the safety of packaged beef and the convenience of detecting, it is necessary to carry out nondestructive detecting directly in the presence of packaging film, without removing the packaging (Jin et al. 2023). Therefore, it is particularly important to rapidly detect meat quality and accurately evaluatei beef quality without destroying the packaging, which are of great significance for monitoring and guaranteeing the quality of packaged raw meat.

The main problem when performing hyperspectral detection in the presence of packaging films is the absorption and scattering of the spectral signal by the packaging film, which can lead to distortion of the detected data (Zhang, Pan, and Lu 2023). Studies have shown that films will be reflected, refracted, scattered, and absorbed in a specific wavelength range, which will affect the final intensity of light received by the sensor (Gowen et al. 2010). Also of interest are the scattering and refraction effects of light in polymer films. It has been noted that PE packaging films reduce the reflectivity of the sample. Therefore, changes in reflectance spectra caused by packaging films should not be ignored, which poses a challenge to HSI non‐destructive detection of packaged beef.

**SCHEME 1:**
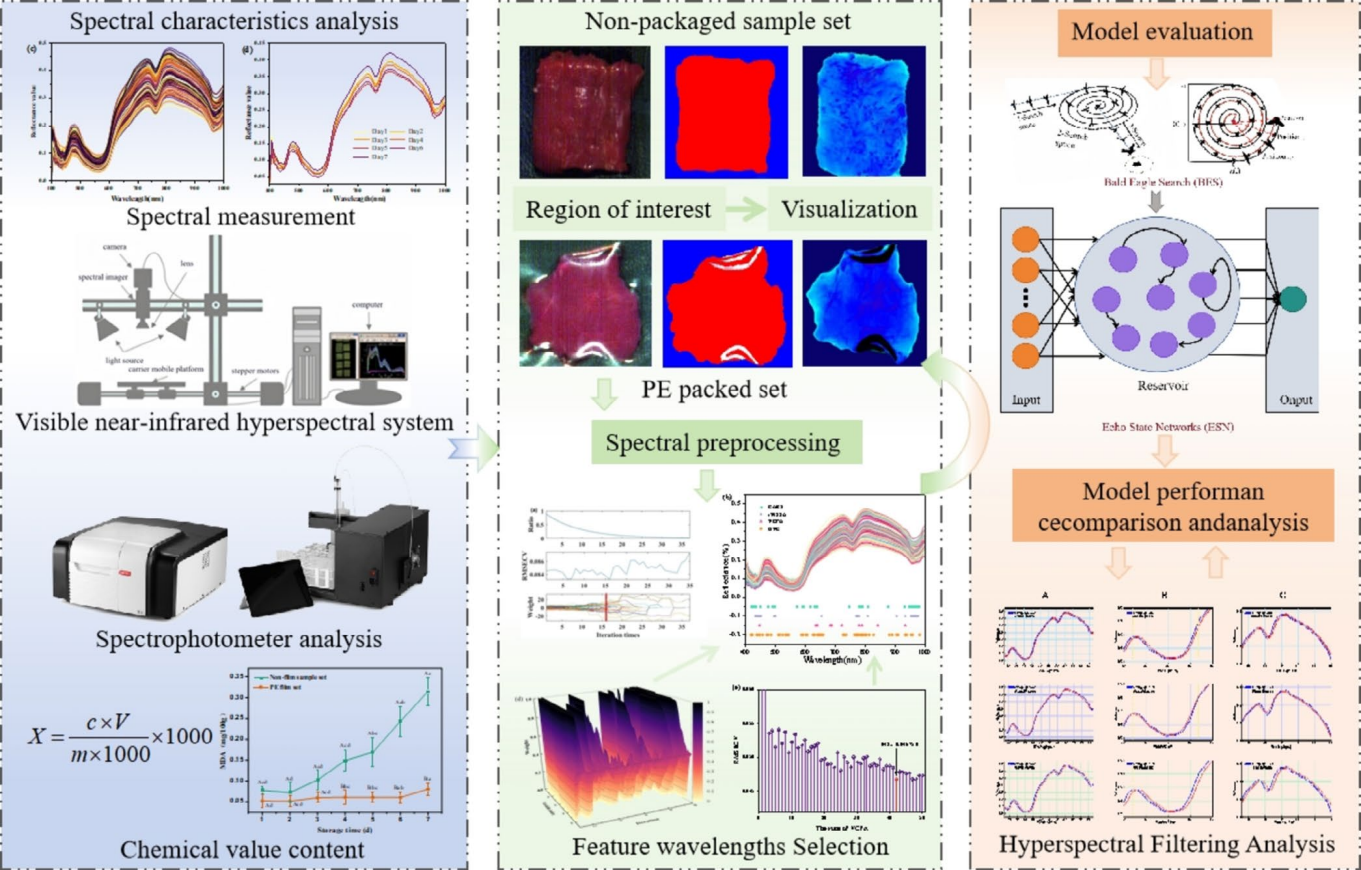
Flowchart of the main steps in the data analyses and model establishment.

In this study, visible near‐infrared hyperspectral (Vis–NIR) imaging technology combined with chemometric methods and deep learning was used to rapidly detect the freshness of PE‐packaged raw beef. Changes in MDA content of beef samples during short‐term storage were analyzed using non‐packaged chilled beef as a control group. A quantitative prediction model of MDA content in raw beef based on different signal characteristics was proposed. On this basis, Gaussian filter, median filter, and finite impulse response filter algorithms were used to optimize the quantitative model for freshness indexes of PE‐packaged beef, so as to improve the prediction accuracy of the model. Rapid detection of the meat quality and accurate evaluation of the beef quality without destroying the package provide a new technical means to realize the rapid detection of packaged meat quality (Scheme 1).

## 2 Materials and Methods

### 2.1 Packaging Films and Samples

In this study, commonly used PE film (thickness 0.04 mm) for cold meat was used for vacuum preservation packaging of fresh beef provided by Ningxia Jingmeit Packaging Material Co. TCA (purity > 99%) was purchased from Shanghai Aladdin Biochemical Technology Co. and thiobarbituric acid was provided by Shanghai Sinopharm Group Chemical Reagent Co.

Beef samples were purchased from Ningxia Guyuan Shangnong Bio‐development Science and Technology Co. All beef was sourced from 20 cattle of the same breed with the age of 2 years old. After slaughtering, the beef samples were refrigerated at 4°C for 48 h for acid removal, followed by splitting. The longest dorsal muscle of the cattle carcass was specifically selected for this experiment and was placed in an insulated box with ice packs and transported to the Meat Processing and Quality and Safety Control Laboratory of Ningxia University, where all the samples were stored in a frozen environment at −88°C.

Prior to the start of the experiment, the knives, plates, and trays used in the experiment were disinfected by wiping with alcohol, followed by irradiation with UV light for 45 min to further sterilize them. During the experiment, the fascia and fat were first removed from the whole fresh beef, and the meat samples were uniformly cut into small pieces with dimensions of 2 cm × 2 cm × 2 cm and a mass of 10 g. All samples were divided into two groups: one group was vacuum‐packed using PE packaging film as the PE packaging group, and the other group was placed on a tray without packaging film as the blank control group. Then, the two groups of prepared beef samples were placed in a constant temperature and humidity chamber at 4°C for 1, 2, 3, 4, 5, 6, and 7 days, respectively. Thirty meat samples from each group were taken daily for hyperspectral measurement and determination of MDA content in a spectral analysis laboratory, and three parallel tests were performed for each determination to ensure the accuracy of the data.

### 2.2 Hyperspectral Imaging System

**FIGURE 1:**
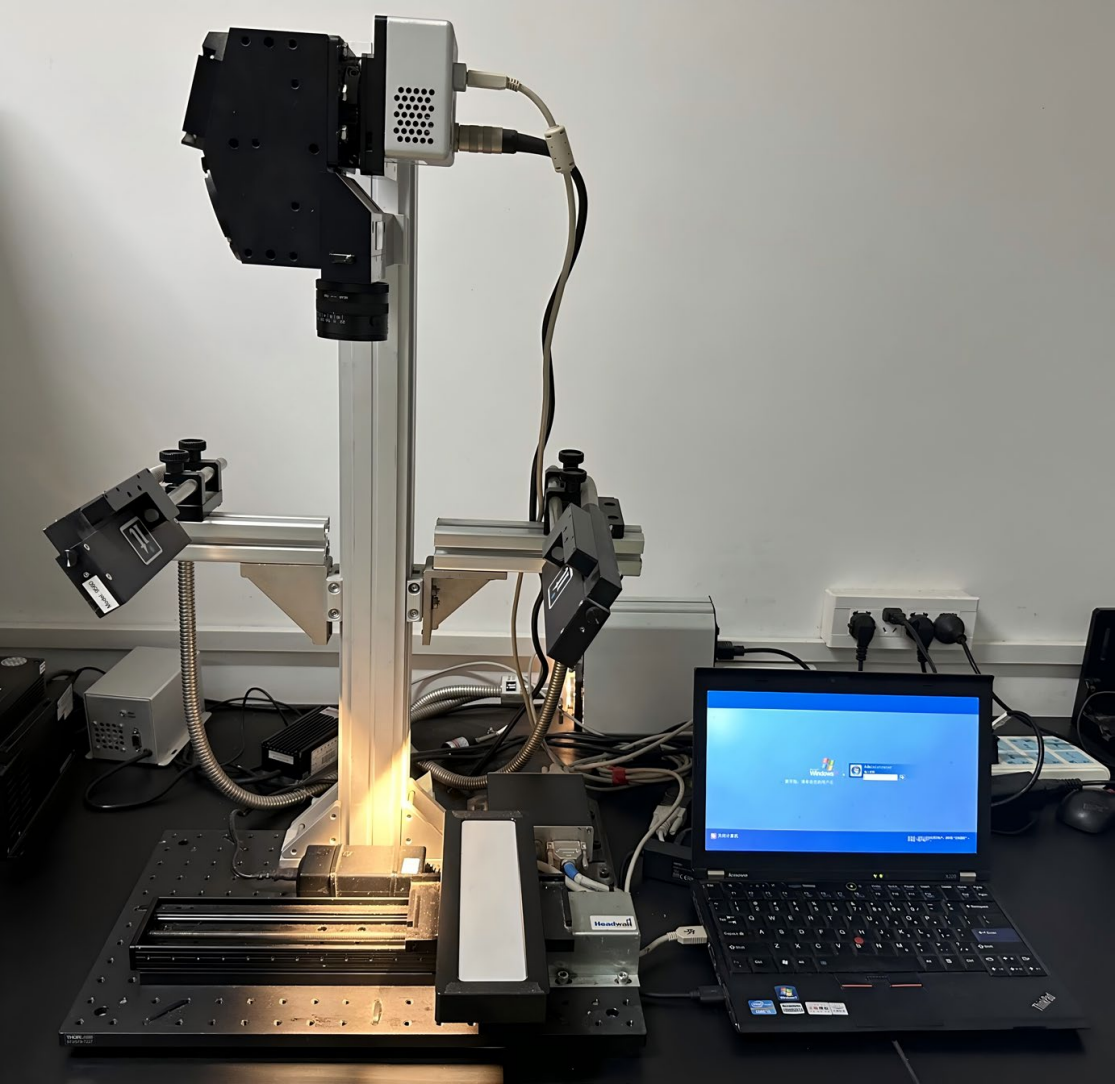
Hyperspectral image acquisition device.

In this study, a Vis–NIR imaging system (Hyper Spec VNIR V10E‐QE) manufactured by Spemicm, Finland, was used to collect spectral data. As shown in Figure 1, the HSI system consists of two major parts: the software control and the hardware device. The software control part is mainly responsible for setting the acquisition parameters and performing the black‐and‐white correction of the images, including system settings, spectral scanning, and image processing. The hardware device is used to scan and collect hyperspectral images of the samples, which mainly includes components such as a spectral imager, light source, camera, lens, and carrier mobile platform, etc. The camera is capable of capturing 125 wavelengths.

The camera is capable of capturing 125 wavelengths, with a spectral acquisition range of 400~1000 nm and a spectral resolution of 5.8 nm. The camera has a sampling spacing of 1.5 nm, a start position of 0.65 cm, a scanning distance of 1.0 cm, a forward speed of 0.15 cm/s, a backward speed of 0.20 cm/s, an exposure time of 6.2 ms and a gain setting of 5. To eliminate the risk of the camera's sensors, the camera is used to acquire images with a spectral resolution of 5.8 nm. In order to eliminate the dark current effect of the camera sensor and the uneven illumination, the instrument was warmed up for 30 min before sample acquisition (Feng et al. 2024). Subsequently, the original hyperspectral images were corrected for black and white with the following formula:

(1) $$I\left(\%\right)=\frac{R-R_{1}}{R_{2}-R_{1}}\times 100\%$$

where *I* is the acquired calibration image, *R* is the raw data, *R*_1_ is the dark reference image produced by covering it with an opaque lens cap, and *R*_2_ is the white reference image produced by scanning a rectangular PTFE plate.

The chemical indicators of beef samples were measured immediately after hyperspectral image acquisition.

### 2.3 MDA Values Measurement

In this study, the spectrophotometric method was used for the determination of MDA in foodstuffs. Firstly, the beef sample was minced, 5 g of minced meat was weighed into a 100 mL conical flask, 50 mL of trichloroacetic acid was added, and the sample was shaken for 30 min and cooled. After that, the sample was filtered through double‐layer quantitative slow filter paper, and 5 mL of the filtrate and the standard solution were put into 25 mL colorimetric tubes. After adding aqueous thiobarbituric acid, the absorbance was measured (Xie et al. 2015), after the reaction at 90°C for 30 min. The content of MDA was calculated using the standard curve, the *X* (mg/kg) was calculated by the following formula:

(2) $$X=\frac{c\times V}{m\times 1000}\times 1000$$

where *C* represents the MDA concentration (in μg/mL) obtained from the standard curve, V is the constant volume of the specimen solution (in mL), m is the mass of the specimen (g) represented by the final specimen solution, and 1000 is the conversion factor.

### 2.4 Multivariate Data Processing

- Extraction of regions of interest: The thresholding method was used to extract the region of interest (ROI) of the sample using ENVI 4.8 software. Firstly, the low reflectance band image was subtracted from the high reflectance band image to highlight the target information and facilitate the setting of the threshold value. Subsequently, an appropriate threshold is set to separate the sample from the background, and a threshold of 0.2 is applied to generate a binarized image, which is filled to eliminate edge noise. Finally, the ROI function is utilized to extract the average spectrum as the spectral value of the sample (Cui, Zhang, et al. 2024).
- Elimination of outliers and division of the sample set: After extracting the spectral data, the Monte Carlo (MC) method was used to reject the anomalous samples (Hao et al. 2023). Subsequently, the joint x and y distance (SPXY) algorithm, which calculates the joint x and y distance between samples, was employed to assess the similarity between data points. The sample set was then divided into calibration and validation sets according to the number ratio of 3:1, and finally 114 calibration samples and 39 validation samples were obtained.
- Data preprocessing: The process of acquiring raw spectral information is often accompanied by some dark current noise, light scattering, and instrumental baseline drift, which may interfere with the accuracy of spectral data (Li et al. 2024). In order to reduce the influence of these interfering factors on the validity of the model and to fully explore the potentially valid information in the spectral data, five mathematical methods, namely, savitzky–Golay smoothing (S‐G), normalization (Nor), multiplicative scatter correction (MSC), baseline, and standard normal variates (SNV), were used to preprocess the spectral data before the model construction.
- Extraction of characteristic wavelength: On the basis of data preprocessing, the characteristic wavelengths were extracted from the whole wavelengths. In order to reduce the dimensionality of the spectral data, simplify the complexity of the model, and improve the prediction accuracy, four variable extraction methods were used to extract the characteristic wavelengths that best represent the spectral characteristics of the samples (Li et al. 2023). The characteristic wavelength extraction methods used in this study include variable combination population analysis (VCPA), competitive adaptive reweighted sampling (CARS), and the interval variable iterative space shrinkage approach (iVISSA).

### 2.5 Modeling Approaches and Evaluation

In this study, a quantitative prediction model for MDA content was constructed using partial least squares regression (PLSR) and an echo neural network (BES‐ESN) optimized by the vulture optimization algorithm. The coefficients of determination (*R* ^2^_C_ and *R* ^2^_P_) and root mean square error (RMSEC and RMSEP) of the calibration and prediction sets were used to assess the accuracy and predictive ability of the models. An accurate predictive model should have a high RPD value, *R* ^2^ close to 1, a low RMSE value, and a small difference between RMSEC and RMSEP (Feng et al. 2024).

Partial least squares regression is a statistical modeling technique used to predict relationships between predictor and response variables in high‐dimensional datasets (Yan et al. 2024). The method not only deals with the multicollinearity problem between variables but also extracts the most important information from the predictor variables, thus achieving effective data reduction and feature extraction while providing robust prediction performance (Lv et al. 2023).

**FIGURE 2:**
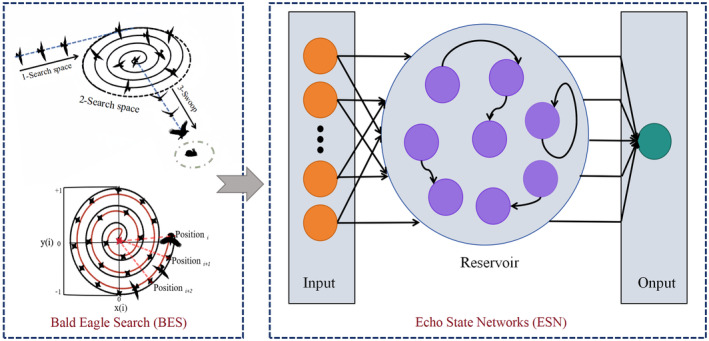
Main technical flow chart. (a) The three main stages of BES hunting; (b) Bald eagles searching within a spiral space; (c) General architecture of an Echo State Network (Koloko et al. 2022).

Bald Eagle Search (BES) is a population optimization algorithm inspired by the predatory behavior of bald eagles in nature. BES efficiently solves high‐dimensional and nonlinear optimization problems by simulating the search and predatory processes of bald eagles (Alsattar, Zaidan, and Zaidan 2020). The introduced variable structure error system contains dynamic adjustment rules that can adapt to the changes of system characteristics during the prediction process (Nicaire et al. 2021). The algorithm shows superiority in several fields with its powerful global search capability and fast convergence speed.

Echo State Networks (ESN) are a specific type of recurrent neural network suitable for processing time series data. The unique features of ESN are its sparsely connected internal neurons and “echo” states, which give the network good memory and dynamic response properties (Khodabandehlou and Fadali 2017). The states of the hidden layer nodes are determined by the inputs, weight matrices, and randomly initialized fixed state transfer matrices, and the external inputs only affect the outputs but not the internal states (Zhang et al. 2020). Combining the prediction ability of ESN with the adaptivity of BES, multiple input variables can be synthesized to generate a single prediction value, thus improving the learning efficiency and prediction accuracy of the whole network. The networt structure of BES‐ESM is shown in Figure 2.

### 2.6 Visualization

Hyperspectral imaging combines image and spectral techniques to produce a hyperspectral three‐dimensional cube, called a hypercube. HSI has a spectral dimension (wavelength axis) and two spatial dimensions (X and Y axes). The visualization map is made by converting the predicted value of each pixel to a different color value through a linear color bar, with similar spectral features having similar colors (Shi, Wang, Hu, et al. 2023). The HSI has a spectral dimension (the wavelength axis) and two spatial dimensions (the X‐axis and the Y‐axis). During the visualization process, the spectrum of each pixel is extracted and stored as a 3D data matrix. The optimal hyperspectral data prediction of the beef samples to be visualized was used as input to the model to obtain the predicted MDA content at each pixel location of the hyperspectral image. Finally, pseudo‐coloring techniques were used to visualize the predicted data on the image at the corresponding pixel locations to generate a visualization map of the sample.

### 2.7 Hyperspectral Filtering Analysis

#### 2.7.1 Gaussian Filtering

A Gaussian spatial filter is a linear filter based on the characteristics of the Gaussian function, which is widely used in image processing. Its main function is to smooth the image to reduce the effect of random noise (Teng et al. 2016). When eliminating the effect of packing film on spectral data, the Gaussian spatial filter actually utilizes its smoothing property to reduce or eliminate the noise or interference introduced by the packing film in the spectral signal.

The working principle of Gaussian filtering is to assign the weight defined by the Gaussian function to the neighborhood of each point in the spectral data and then calculate the weighted average as the post‐filtered value for that data point (Poroshin, Bogomolov, and Lysenko 2017). For the specific operation process, suppose that there is a set of one‐dimensional data *d*. For any data point *d*[*i*] in *d*, its post‐filtered value *G*[*i*] can be calculated by the following formula:
(3)

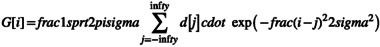

where *G*[*i*] is the filtered data; *d*[*j*] is the original data point; (*i*, *j*) defines the position of the data point; and *sigma* is the standard deviation of the Gaussian function. This value determines the weight of the data point with its neighboring points. The larger its value is, the points farther away from the current data point are given a larger weight; conversely, the smaller its value is, only some data in the neighborhood of the current data point are given a larger weight.

#### 2.7.2 Median Filtering

The Median filter is a nonlinear filter commonly used in denoising operations in image processing (Morillas, Gregori, and Sapena 2011). The median filter works as follows: for each pixel position of (*i*, *j*) in an image, the surrounding pixel points of this pixel in a defined region are first considered. Next, all these pixel points are sorted according to their gray level. Then, the median value is selected from them as the gray level of that pixel point after filtering. Thus, median filtering is actually a nonlinear smoothing process (Ye 2011). For one‐dimensional signals, it can be calculated by the following formula:

(4) $$y\left[n\right]=\text{median}\left(x\left[n-K:n+K\right]\right)$$

where *y*[*n*] is the output sequence, i.e., the filtered result; *x*[*n*] is the input sequence, i.e., the original data being filtered; *K* is half of the filtering window, and if the size of the window is such that the median filter examines the current point as well as the *K* samples before and after it at each moment; and *n* corresponds to a one‐dimensional signal when denoting a temporal or spatial sequence.

#### 2.7.3 Finite Impulse Response Filtering

A Finite impulse response filter (FIR) is a common type of filter in digital signal processing, which is mainly characterized by having a finite response time and a fixed input/output delay (Pinheiro, Petraglia, and Petraglia 2018). By selecting these impulse response coefficients, different types of filters can be implemented to remove noise, emphasize specific frequency bands, or achieve other signal processing purposes (Ling et al. 2015). The mathematical expression for FIR filters is given below:

(5) $$y\left[n\right]=\sum_{i=0}^{N}h\left[i\right]\cdot x\left[n-i\right]$$

where $y\left[n\right]$ is the output sequence; $x\left[n\right]$ is the input sequence; and $h\left[i\right]$ is the impulse response of the filter. For a FIR filter of length *N* + 1, there are a total of *N* + 1 coefficients.

In this study, all procedures, including dataset partitioning, image processing, model construction, spectral preprocessing, and characteristic wavelengths extraction are performed in Matlab R2018b software.

## 3 Results and Discussion

### 3.1 Measurement Results of MDA

**FIGURE 3:**
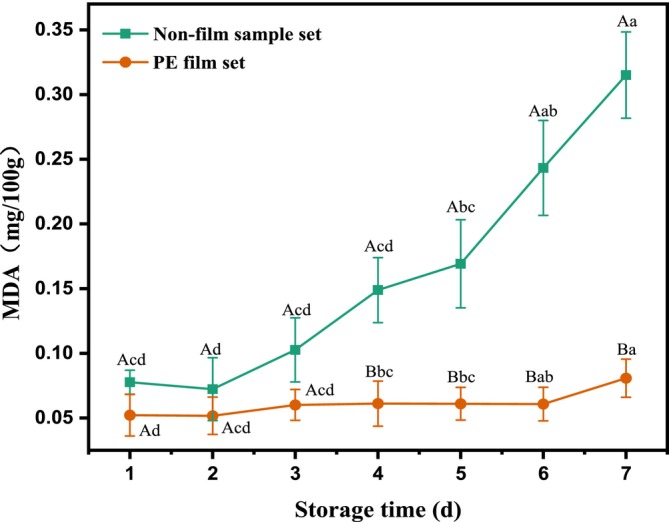
Variation trend of MDA during storage of raw beef in non‐packaged and PE packaging.

Malondialdehyde is a lipid oxidation product commonly found in meat and other foods and is often used as a key indicator of the degree of lipid oxidation in foods. As shown in Figure 3, the MDA values of beef samples from the blank group showed an increasing trend with the increase of storage time. This phenomenon indicated that fat oxidation led to deterioration of muscle texture, bad odor, and rancidity with the increase of storage time, thus reducing the freshness and nutritional quality of the meat (Xie et al. 2015). During the first 3 days of storage, the mean value of MDA content of raw beef was < 0.15 mg/100 g, and no spoilage samples were observed. By the 4th day, the MDA content increased significantly (*p* < 0.05) and the mean value was more than 0.15 mg/100 g, indicating that the beef samples showed signs of spoilage. Compared with the blank control group, the MDA content of fresh beef packed in PE packaging showed a slow increase with storage time, and the change was not significant (*p* > 0.05), indicating that the vacuum packaging significantly delayed the oxidization process of the beef and improved the preservation effect. During the storage period, the mean value of MDA of PE‐packed beef was always lower than 0.15 mg/100 g, and no spoilage occurred. This proved that PE vacuum packaging could effectively reduce the contact of oxygen with beef, thus slowing down the oxidation process and prolonging the freshness period of meat.

### 3.2 Analysis of Spectral Characteristics

**FIGURE 4:**
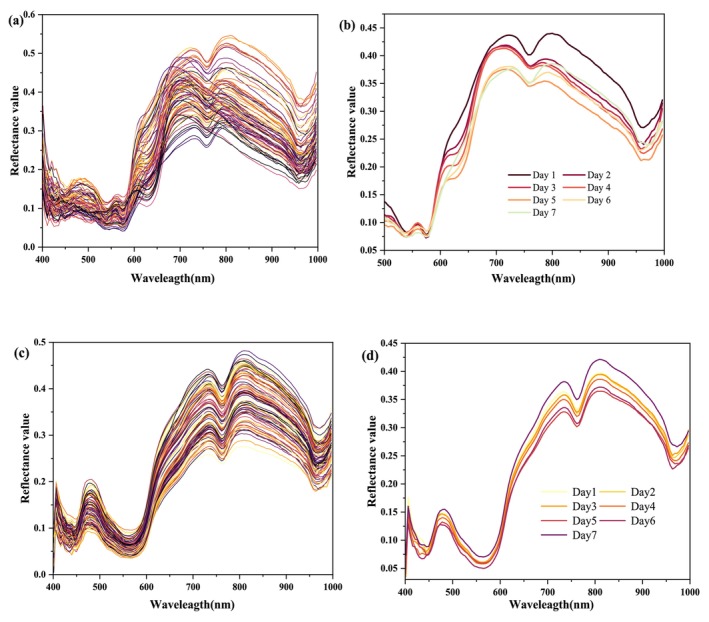
The visible near‐infrared spectral curves of beef samples during storage. (a) The original spectral curves of all beef samples in the blank control group during storage; (b) the average spectral curves of beef samples with different storage times in the blank control group; (c) the original spectral curves of all PE‐packaged beef during storage; and (d) the average spectral curves of PE‐packaged beef for different storage times.

Observation of the original spectral curves shows that the spectral shape features are similar, the peaks of the spectral curves have the same trend, and the spectral reflectance is in the range of 0.3–0.55. Figure 4a shows the important absorption peaks in the wavelength range of 400–1000 nm. The absorption peak observed at 560 nm may be related to respiratory pigments, mainly myoglobin or deoxymyoglobin (Guo et al. 2010). The band around 610 nm is the third‐order frequency doubling absorption peak of the amino group, and 739 nm is the third‐frequency doubling region of –CH (Dong et al. 2022). The band related to the third‐frequency doubling of the O–H stretching or the myoglobin oxidation around at 760 nm. The band at 810 nm is the vibrational multiplier absorption of C–H bonds in proteins (Cui, Li, et al. 2024). And the 960 nm wavelength is the second multiplicative frequency of O–H stretching due to water (Tang et al. 2024). In addition, different trends were observed for small portions of the spectral profile and the overall spectral curves. For example, absorption peaks appeared at 600–650 nm and around 700 nm and disappeared at 810 nm, which could be attributed to the changes in beef quality with increasing storage time, changes in some physical characteristics and chemical compositions, such as loss of water, changes in tissue color, proteolysis, and oxidation of fats, which led to changes in spectral absorption (Dong et al. 2024).

It can be observed that the reflectance of beef decreased and then increased with storage time in Figure 4b. The spectral shapes of the average reflectance spectral curves of beef samples under different storage times were similar in the whole wavelength range of 400–1000 nm. However, with the extension of storage time, the reflectance of the characteristic peaks decreased and then increased. The reflectance of the average spectral curve decreased with the extension of storage time in the first 6 days, and then increased in the spectral curve on the 7th day. Preliminary analysis of the above phenomenon may be related to the mutual transformation of myoglobin during the storage period. At the early stage of storage, the content of oxygenated myoglobin and high iron myoglobin of raw fresh beef changed consistently, which made the meat color inclined to dark red and the reflectance decreased. With prolonged storage, the change in oxygenated myoglobin exceeded that of high iron myoglobin, resulting in a change in beef color to bright red and an increase in reflectance.

From Figure 4c, it was observed that the peak trends of all spectral curves were basically the same, indicating that the packaged beef had a small change in quality during the 7‐day storage period. The spectral reflectance of the full‐band spectral curves of packaged beef was overall < 0.5, which was lower than that of the blank group. This phenomenon may be caused by the effect of transmission and scattering of the packaging film. Because part of the near‐infrared light of the packaging material is reflected, the reflectivity is low. The 610, 739 and 810 nm are related to chemical bonding effects such as –CH_3_, O–H, and C–H. The disappearance of the absorption peak of beef at 560 nm is observed, which may be caused by the influence of chemical substances within the PE packaging film material.

In Figure 4d, the reflectance of beef is shown to gradually decrease as storage time increases. And the overall trend of the spectral curves during storage was consistent, indicating that the quality of the beef did not change much. In the whole wavelength range of 400–1000 nm, the average spectral reflectance change range of PE‐packed beef stored for 7 days was small compared with that of unpacked beef, indicating that color changes in the beef during storage were less pronounced with PE packaging, and the conversion between oxygenated myoglobin and high iron myoglobin occurred at a slower rate.

### 3.3 Multivariate Data Processing

#### 3.3.1 Optimal Preprocessing Methods of Spectra

In order to improve the quality of spectral data, reduce noise interference, and remove the effect of baseline so as to extract useful information, the raw spectral data of PE‐packed beef and blank group beef were preprocessed using the Normalize, Baesline SNV, MSC, Detrend, and OSC preprocessing methods, respectively. The PLSR modeling results are shown in Table 1. Compared with the blank group, the modeling effect of raw spectral data of beef in the PE packing group was lower. This phenomenon may be due to the interference introduced by the PE packaging film, which affects the reflectance and scattering of light, thereby distorting the spectral data. These distortions can complicate the modeling process and make it more challenging to extract accurate information related to MDA content. After preprocessing, the predictive performance of the models for both data groups was better than their original data. There were also significant differences in the prediction of MDA content by different preprocessing methods. Among the preprocessing methods, the baseline and SNV methods consistently led to the best improvements in model accuracy. For the blank control group, SNV resulted in the highest increase in *R* ^2^_P_ (from 0.8295 to 0.8606), demonstrating its effectiveness in reducing scatter effects and normalizing the spectral data. Similarly, the MSC method improved the model by addressing light scattering effects, which led to a more accurate prediction of MDA content. Notably, the Detrending was particularly effective for the PE‐packed beef samples, improving prediction accuracy by reducing baseline shifts. For the blank group spectra, the *R* ^2^_P_ of all the preprocessing and prediction models was improved, and the RMSEP was < 0.1, except for the OSC and Detrend methods, which indicated that the preprocessing methods predicted the beef MDA values better. The highest model accuracy was obtained by using the SNV spectral pretreatment method (*R* ^2^_C_ = 0.8885, RMSEC = 0.5313, *R* ^2^_P_ = 0.8606, RMSEP = 0.4681). For the PE packing group beef, after preprocessing, the performance of the prediction set was improved after processing by all methods except the OSC method. This may be due to the fact that OSC improves the model by removing variation that is not relevant to the response variable, and improper parameter settings may incorrectly remove important information that contributes to the model, resulting in a decrease in model performance. Among the other preprocessing methods, the Detrend method processed the best modeling results (*R* ^2^_C_ = 0.7292, RMSEC = 0.6382, *R* ^2^_P_ = 0.6805, RMSEP = 0.6571). In addition, the PLSR modeling effect of the best preprocessing method was observed for both data sets, and the prediction effect of the PE‐packed set was lower than that of the blank set.

**TABLE 1:** Results of prediction models for MDA content in non‐packaged and PE packaged beef based on different pretreatment spectral data.

Sample source	Data processing method	Calibration		Prediction	
		*R* ^2^_C_	RMSEC	*R* ^2^_P_	RMSEP
Non‐packaged beef sample	Raw	0.8516	0.5357	0.8295	0.5812
	Normalize	0.8791	0.5325	0.8283	0.5819
	Baesline	0.8926	0.4987	0.8561	0.4787
	SNV	0.8885	0.5313	0.8606	0.4681
	OSC	0.8012	0.5992	0.7741	0.6019
	Detrend	0.8211	0.5879	0.7981	0.6073
	MSC	0.8647	0.5342	0.8482	0.5649
PE packed beef sample	Raw	0.6441	0.6606	0.5977	0.6978
	Normalize	0.6856	0.6531	0.6638	0.6593
	Baesline	0.7446	0.6069	0.6697	0.6604
	SNV	0.7266	0.6395	0.6278	0.6877
	OSC	0.6431	0.6692	0.5993	0.7201
	Detrend	0.7292	0.6382	0.6805	0.6571
	MSC	0.6978	0.6499	0.6234	0.6885

#### 3.3.2 Identification of Feature Wavelengths

**FIGURE 5:**
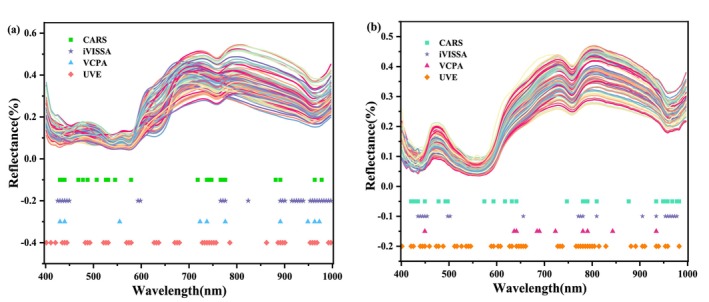
The distribution of characteristic wavelengths of beef samples in different groups in the whole band: (a) unpacked beef samples; (b) PE‐packed beef samples.

The large amount of redundant information contained in the 125 bands of the full spectrum can lead to complexity and long time consumption for computer model training. Four feature wavelength selection methods (CARS, iVISSA and VCPA) are used to reduce the hyperspectral data dimensions and provide a more reliable database for subsequent analysis and application. The distribution results of the characteristic wavelengths are shown in Figure 5.

**FIGURE 6:**
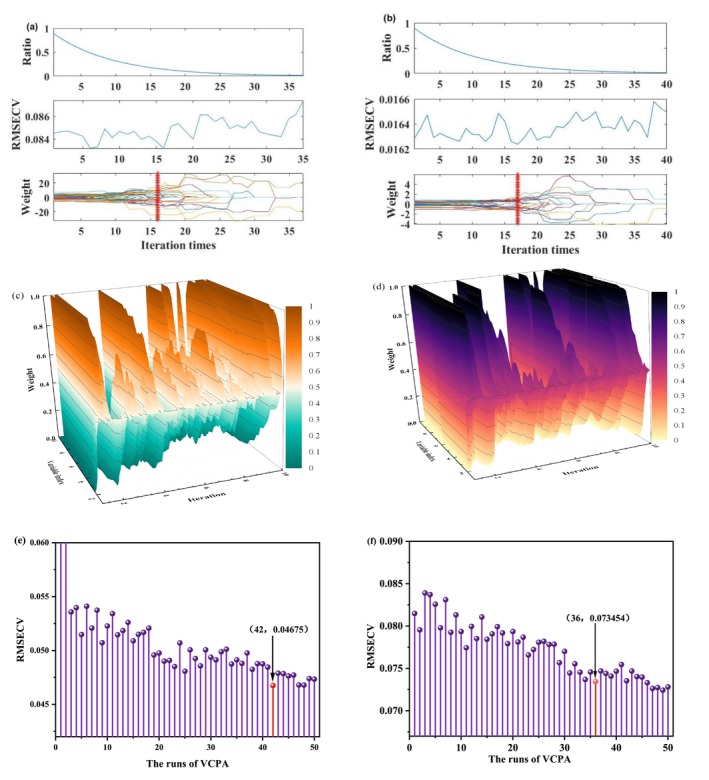
Characteristic wavelengths related to MDA content in beef under the blank group (left) and in the PE packaging group (right).

When the CARS method was used to extract the characteristic wavelength of MDA content in unpacked and PE‐packed beef (Figure 6a,b), to ensure the stability of the results and the representativeness of the extraction characteristic wavelength, the parameters were set as 500 times of sampling, the data processing mode was “center,” the number of principal components (PCs) was 20, and the number of cross‐validations was 20 (Lv et al. 2023). The results showed that the number of characteristic wavelengths related to MDA content in non‐packaged beef and PE‐packed beef samples was 43 and 26, accounting for 34.4% and 20.8% of the whole band, respectively. The extraction of characteristic wavelength improved the computational efficiency and prediction accuracy of the model while reducing data redundancy. The specific characteristic wavelengths positions are shown in Figure 5. The CARS algorithm evaluates the contribution of each wavelength through multiple samples, gradually eliminates the wavelengths with weak relationships with the target variable, and finally selects the most representative wavelength for MDA prediction.

The iterative process of extracting characteristic wavelengths related to MDA content in non‐packaged and packaged beef using the iVISSA method is shown in Figure 6c,d. The parameters of the iVISSA algorithm were set to a maximum principal component score of 20, with f and 10 iterations. The iVISSA algorithm gradually screens out wavelengths with significant differentiation and gradually optimizes the wavelength selection process, thus improving the predictive ability of the model (Feng et al. 2024). The number of characteristic wavelengths in the non‐packaged group was obtained as 32, and the number of characteristic wavelengths in the packaged group was 27, accounting for 25.6% and 21.6% of the full wavelengths, respectively. It can be observed from Figure 5 that characteristic wavelengths extracted by the iVISSA method not only reduce redundant information but also retain the key wavelengths that are closely related to the MDA content in beef.

Figure 6e,f show the distribution of RMSECV during the sampling process of the VCPA algorithm. Running the VCPA algorithm uses a maximum principal component of 10 and a BMS sampling of 1000. The wavelength screening run for beef MDA content in the blank group, and the minimum value of the RMSECV was achieved when the number of runs was 42. The wavelength screening run of MDA content of beef in the PE packaging group, when the number of runs was 36, had the minimum value of 0.73454 obtained by RMSECV.

### 3.4 Modelling Analysis of the Modeling Results

The extracted characteristic wavelengths provide a basis for further predictive modeling of beef freshness and oxidation. The PLSR model and BES‐ESN model were developed based on full spectra and characteristic wavelengths extracted by different methods, and the results are shown in Table 2. Both models showed good performance for the prediction of MDA content in unpacked and PE‐packed beef. Specifically, the BES‐ESN model demonstrated higher predictive accuracy, with *R* ^2^_C_ of 0.9201 and *R* ^2^_P_ of 0.9207 for the unpacked beef samples, compared to 0.8561 (*R* ^2^_C_) and 0.8482 (*R* ^2^_P_) for the PLSR model. Similarly, for PE‐packed beef, the BES‐ESN model achieved an *R* ^2^_C_ of 0.8166 and *R* ^2^_P_ of 0.8131, which was higher than the PLSR model that had an *R* ^2^_C_ of 0.7292 and *R* ^2^_P_ of 0.6805. This indicates that the BES‐ESN model, particularly when combined with optimization algorithms, is better suited for handling complex, non‐linear relationships in the spectral data. And it could be clearly found that the overall performance of the BES‐ESN models was better than that of the PLSR models, showing more potential and was expected to play an active role in the evaluation of MDA content. The extraction of characteristic wavelengths significantly reduced the number of wavelengths, and the performance of the model based on characteristic wavelengths for the two data sets was mostly better than that of the respective full‐spectra models (Li et al. 2024).

**TABLE 2:** Modelling results of the full band and feature wavelength based on PLSR and BES‐ESN.

Data sets	Method	No. of variables	LVs	PLSR				BES‐ESN			
				*R* ^2^_C_	RMSEC	*R* ^2^_P_	RMSEP	*R* ^2^_C_	RMSEC	*R* ^2^_P_	RMSEP
Non‐packaged sample set	Raw	125	11	0.8516	0.5357	0.8295	0.5812	0.9005	0.1903	0.9027	0.1887
	CARS	22	12	0.9151	0.4725	0.9084	0.4763	0.9257	0.1608	0.9201	0.1681
	iVISSA	21	12	0.8704	0.4636	0.8583	0.4774	0.9207	0.1741	0.8203	0.2226
	VCPA	10	10	0.8113	0.5983	0.8167	0.5902	0.8982	0.1998	0.9105	0.1809
PE packed sample set	Raw	125	10	0.6441	0.6706	0.5977	0.6978	0.8198	0.2231	0.8166	0.2126
	CARS	21	10	0.7219	0.6409	0.7315	0.6381	0.8127	0.2249	0.8131	0.2237
	iVISSA	28	10	0.7013	0.6189	0.6969	0.6191	0.7971	0.2316	0.7151	0.2352
	VCPA	11	8	0.7744	0.5902	0.7667	0.6196	0.7858	0.2293	0.8298	0.2203

By comparing the modeling results of the four characteristic wavelengths, the results of the blank group with wavelengths extracted by CARS and iVISSA were better. The VCPA method selected a lower number of characteristic wavelengths than that of iVISSA and CARS, but the modeling results were not good. It may be due to the fact that part of the important information related to MDA content was lost in the process of extracting characteristic wavelengths. Comprehensive analysis showed that the CARS‐BES‐ESN model in the blank group had the best performance, with *R* ^2^_C_ of 0.9257, *RMSEC* of 0.1608, *R* ^2^_P_ of 0.9201, and *RMSEP* of 0.1681. The BES‐ESN model based on the extraction of feature wavelengths by VCPA in the PE packaging group had the best results, with an *R* ^2^_P_ of 0.8298 and an *RMSEP* of 0.2203, and the prediction set was greatly improved. It probably be by combining and projecting the original variables to construct new features, which effectively capture the important information in the data. The other model results had lower prediction sets compared to the predictions of the full‐spectral model, possibly due to the extraction of wavelengths eliminating important information bands, resulting in lower model results.

Overall, within the near‐infrared region, the summarized analysis showed that the characteristic wavelengths could effectively improve the modeling results, and the model performance of PE‐packed beef samples was usually less desirable than that of the unpacked beef sample group. It indicated that the spectral characteristics of PE packaging film might affect the accuracy of quantitative prediction modeling in hyperspectral nondestructive detecting of PE‐packed raw meat. For the unpacked group, the higher number of characteristic wavelengths may reflect that its spectral information was more complex and was more affected by external oxidizing factors, while the PE‐packed group had relatively simple characteristic wavelengths due to the protection of the packaging, and thus fewer feature wavelengths were extracted. Therefore, more methods need to be explored to correct the hyperspectral data of packaged samples to enhance the accuracy of the model.

### 3.5 Optimisation of PE Packaging Beef Model

In this study, three different filtering algorithms were used to correct the reflectance spectral data of PE‐packaged beef. Based on the corrected reflectance spectra, the pre‐filter optimal modeling approach was applied to develop models for predicting MDA content in packaged beef. The prediction results of these models were compared with the results before optimization to determine the optimal modeling method.

#### 3.5.1 Spectral Analysis Based on Filter Correction

**FIGURE 7:**
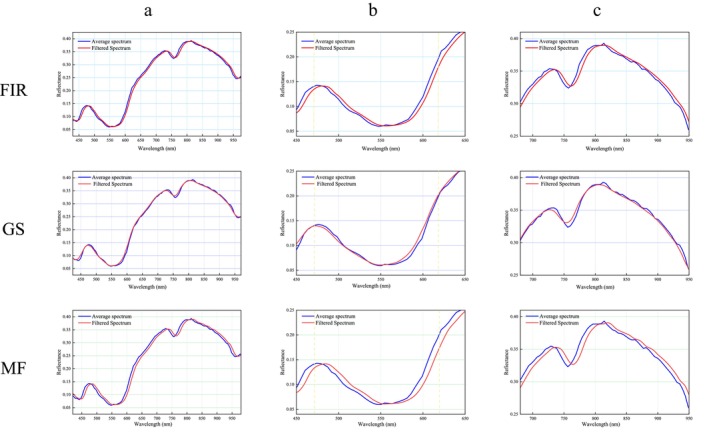
Average spectra before and after filtering by FIR, MF, and GS filtering methods (a) full band; (b) local amplification of: 480–620 nm; (c) local amplification of 650–850 nm.

In general, the packaging material causes some of the NIR light to be reflected directly back to the fiber optic probe, thus significantly affecting the reflectance of the NIR spectrum. In addition, the porous structure of the packaging material causes the light to form complex and diverse optical paths when penetrating the sample surface. The results show that packaging significantly interferes with the NIR reflectance and spectral shape of beef samples. The packaging changes the scattering properties and optical range of light, causing light to scatter and diffuse between the fiber probe and the sample, resulting in many spurious peaks. In this study, different spectral filtering methods were applied to the NIR spectra of PE‐packed beef to compare their potential to reduce packaging spectral interference. Figure 7 shows the average spectra before and after filtering by FIR, MF, and GS filtering methods for full band and local amplification. It can be seen from the full wavelength band that three filtering methods were effective in correcting the spectral scattering interference of packaged beef and eliminating some of the unwanted signals. It can be seen from Figure 7b,c localized bands, the FIR and MF methods were less effective in correcting some of the packaging‐induced spectral interferences uncorrected, while the GS method was more effective. Therefore, GS filtering is more suitable for eliminating the interference of packaging materials on beef NIR spectra in terms of spectral feature reproduction.

#### 3.5.2 Quantitative Prediction of Spectral Filter Correction

The original spectral data, the optimal preprocessing data, and the optimal characteristic wavelength data of PE‐packed beef were corrected based on the three filtering algorithms of MF, GS, and FIR, respectively. The corrected data were compared with the original modeling data, and the modeling results are shown in Table 3. It was found that the *R* ^2^_P_ of the original data corrected by the three processing methods of MF, GS, and FIR increased by 0.0549, 0.0634, and 0.0051, respectively, with the highest prediction accuracy after GS filtering (*R* ^2^_P_ = 0.6611, *RMSEP* = 0.2467). This indicates that the GS method provided the most significant improvement in model performance. These improvements suggest that GS filtering was the most effective in reducing spectral noise and interference, particularly from the packaging material, leading to more accurate MDA content predictions in the PE‐packed beef. After the Detrend preprocessing of the spectral data corrected by the three filtering methods, it was found that only the data processed by GS were improved (*R* ^2^_P_ = 0.7509, *RMSEP* = 0.2376), which might be due to the fact that the combination of the other two filtering methods with Detrend eliminated part of the important information, resulting in a limited improvement of the prediction accuracy (Teng et al. 2016). After extracting the characteristic wavelengths from the Detrended preprocessed data by VCPA, it was found that only the *R* ^2^_P_ of the GS‐passed data was improved compared to the data without correction characteristic wavelengths, and there was little change in the other two methods. The three model combinations after extracting the characteristic wavelengths were compared with the original data, and all of them obtained satisfactory the predictive results, among which the BES‐ESN model of GS‐Detrend‐VCPA was the best (*R* ^2^_C_ = 0.8798, *RMSEC* = 0.1951, *R* ^2^_P_ = 0.8309, *RMSEP* = 0.2180). The results showed that prediction effect of NIR spectra based on different filtering methods on MDA content in beef was better than that of the unfiltered NIR spectra, which highlighted the importance of spectral filtering in improving the performance of NIR models in predicting quality parameters of PE‐packed meat.

**TABLE 3:** MDA modelling results of packaged beef based on different filter treatments.

Filtering methods	Variable combination methods	LVs	Calibration set		Prediction set	
			*R* ^2^_C_	RMSEC	*R* ^2^_P_	RMSEP
Raw	Raw	6	0.6441	0.6706	0.5977	0.6978
	Detrended	8	0.7292	0.6382	0.6805	0.6571
	Detrend‐VCPA	8	0.7858	0.2293	0.8298	0.2203
MF	Raw	10	0.7971	0.2361	0.6526	0.2496
	Detrended	8	0.8166	0.2114	0.6809	0.2412
	Detrend‐VCPA	8	0.7830	0.2395	0.7051	0.2384
GS	Raw	8	0.7780	0.2301	0.6611	0.2467
	Detrended	8	0.7998	0.2255	0.7509	0.2376
	Detrend‐VCPA	11	0.8798	0.1951	0.8309	0.2180
FIR	Raw	11	0.7278	0.2309	0.6028	0.2511
	Detrended	11	0.8029	0.2182	0.6247	0.2502
	Detrend‐VCPA	12	0.8159	0.2096	0.7309	0.2262

### 3.6 Visualization

**FIGURE 8:**
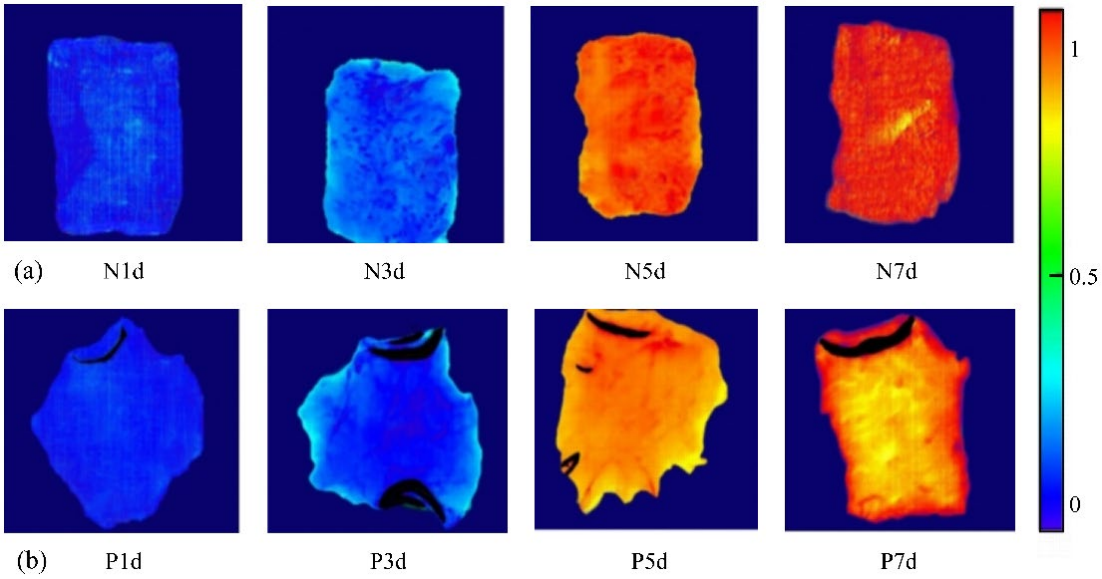
Visualization map of HSI reflectance index for different producing regions. (a) Visualization of non‐packaged samples; (b) Visualization of PE‐packed samples.

One distinctive advantage of HSI lies in its capability for visualization. Based on the optimal model, the linear color scale was applied to visualize the MDA content and distribution within beef samples from different packing groups shown in Figure 8. The background is represented by dark blue, while the sample regions are highlighted in color. The different colors helped to visualize the variations in MDA concentration within the chemical maps. Using the optimal model, a linear color scale was applied to illustrate the MDA content and its distribution within beef samples across various packaging groups. This color differentiation effectively visualizes the variations in MDA concentration within the chemical maps. Following the jet chromaticity band display principle, colors approaching red signify a higher concentration of the measured substance, while colors shifting toward blue indicate a lower concentration (Shi, Wang, Shi, et al. 2023). The chemical map transitions from blue to yellow and ultimately to red, effectively illustrating the increasing trend of MDA content in the beef. The changes in freshness indicators of the samples in the blank group (N1‐7d) were more pronounced than those in the vacuum‐packaged group (P1‐7d). This observation aligns with previous freshness and oxidation assessments, indicating that the freshness preservation of the beef was significantly improved by the vacuum packaging and effectively controlled the rate of lipid oxidation in the beef. Therefore, the visual distribution map enables visualization of the amount of MDA levels within beef samples. Some vertical streaks appeared, likely caused by vibrations during the spectral scanning process (Dong et al. 2025). This distribution map can provide a theoretical foundation for the development of each studied property and can be used for fast and non‐destructive measurements.

## 4 Conclusions

This study demonstrated the effectiveness of HSI combined with chemometrics for evaluating MDA content in both PE‐packed and non‐packed beef. The results highlighted the significant role of PE packaging in maintaining beef quality by slowing lipid oxidation. Preprocessing of spectral data, along with feature wavelength extraction, significantly improved the model's predictive accuracy. In addition, an innovative approach based on the ESN parameters from the BES optimization algorithm allowed for the highlighting of a new tool for the prediction of the MDA content in beef. The SNV‐CARS‐BES‐ESN method is suitable under unpacked conditions with an *R* ^2^_C_ of 0.9257, an *RMSEC* of 0.1608, an *R* ^2^_P_ of 0.9201, and an *RMSEP* of 0.1681. The optimal Detrend‐VCPA‐BES‐ESN model of PE‐packed beef had a good predictability with *R* ^2^_C_ of 0.7858, RMSEC of 0.2293, *R* ^2^_P_ of 0.8298, and RMSEP of 0.2203. Filtering algorithms further enhanced prediction accuracy by correcting spectral interference caused by packaging. The raw data were corrected by the three processing methods of MF, GS, and FIR, and the *R* ^2^_P_ was improved by 0.0549, 1.2588, and 0.0051, respectively. The GS‐Detred‐VCPA‐BES‐ESN model showed the best results (*R* ^2^_C_ = 0.8798, *RMSEC* = 0.1951, *R* ^2^_P_ = 0.8309, *RMSEP* = 0.2180). The results demonstrated the feasibility of using HSI to evaluate the MDA content of beef, even under the condition of PE packaging film, which provided a theoretical basis and data support for realizing nondestructive detection of packaged raw meat quality to ensure meat quality and safety throughout storage and transportation. Future studies could explore the effects of different packaging materials or methods to refine meat preservation strategies. Additionally, further studies could explore the integration of HSI with other advanced sensors or real‐time monitoring systems for continuous quality assessment during the meat supply chain.

## Author Contributions

**Cheng Wu:** conceptualization (lead), methodology (lead), writing – original draft (lead). **Yingjie Feng:** data curation (lead), writing – review and editing (lead). **Songlei Wang:** funding acquisition (lead), project administration (lead), supervision (lead). **Jiarui Cui:** software (lead), supervision (equal), visualization (equal). **Zhang Yao:** investigation (lead). **Hailong Xu:** validation (lead).

## Conflicts of Interest

The authors declare no conflicts of interest.

## Data Availability

The data generated during this study are available from the corresponding author upon reasonable request.
